# Antioxidant Capacity and Proanthocyanidin Composition of the Bark of *Metasequoia glyptostroboides*

**DOI:** 10.1155/2014/136203

**Published:** 2014-03-17

**Authors:** Fengyang Chen, Lin Zhang, Shuling Zong, Shifang Xu, Xiaoyu Li, Yiping Ye

**Affiliations:** Institute of Materia Medica, Zhejiang Academy of Medical Sciences, Tianmushan Road 182, Hangzhou, Zhejiang 310013, China

## Abstract

*Metasequoia glyptostroboides* Hu et Cheng is the only living species in the genus *Metasequoia* Miki ex Hu et Cheng (Taxodiaceae), which is well known as a “living fossil” species. In the Chinese folk medicine, the leaves and bark of *M. glyptostroboides* are used as antimicrobic, analgesic, and anti-inflammatory drug for dermatic diseases. This study is the first to report the free radical scavenging capacity, antioxidant activity, and proanthocyanidin composition of the bark of *M. glyptostroboides*. We observed total of six extracts and fractions, which were easily obtained by water-ethanol extraction and followed by a further separation with D101 resin column chromatography, had significant DPPH radical, superoxide anion radical, and hydroxyl radical scavenging capacity, total antioxidative capacity (T-AOC), lipid peroxidation inhibitory activity, and metal ions chelating capacity. The fraction MGEB, which was obtained by 60% ethanol extraction and followed by a further separation with D101 resin column chromatograph, possessed the highest proanthocyanidin content and the highest free radical scavenging and antioxidant activities. Furthermore, MGEB could significantly protect against CCl_4_ induced acute liver injury through inhibition of oxidative stress in mice. In addition, ten proanthocyanidins were isolated from MGEB, and six of them were firstly reported from this plant.

## 1. Introduction

Oxidation is essential to many organisms for the production of energy to fuel biological processes. However, reactive oxygen species (ROS) are often overproduced under pathological conditions, resulting in oxidative stress. The oxidative damage is a crucial etiological factor implicated in several chronic human diseases, such as diabetes mellitus, cancer, cardiovascular diseases, atherosclerosis, arthritis, and neurodegenerative diseases, as well as in degenerative processes associated with aging [1]. Therefore, the researches on antioxidants, especially exploration of potent natural compounds with low cytotoxicity from plants, have become an important branch of biomedicine [2].

The phenolic compounds, extensively distributed in different parts of the plant, have been proved to be natural antioxidants. The tree bark, which is easily available throughout the year and even the waste residues of some industrial production (such as paper production), is usually rich in phenolic compounds [3–7]. Up to now, the most prominent phenolic antioxidant from tree bark is Pycnogenol, which is commercially extracted from French maritime pine* Pinus pinaster* and mainly composed of proanthocyanidins (from 65% to 75%). In view of its excellentfree radical scavenging capacity and antioxidant activity, Pycnogenol is now utilized throughout the world as a nutritional supplement and as a phytochemical remedy for various diseases ranging from chronic inflammation to circulatory dysfunction, including several impaired psychophysiological functions [8].

*Metasequoia glyptostroboides* Hu et Cheng (dawn redwood, Chinese redwood, or water fir in English and shui shan in Chinese) is the only living species in the genus* Metasequoia* Miki ex Hu et Cheng (Taxodiaceae), which is well known as a “living fossil” species. The discovery of the first living* M. glyptostroboides* in the south of China in the early 1940s was one of the greatest botanical discoveries of the 20th century [9]. Now, it is mainly used as landscape tree and propagated in many parts of China and other countries. In the Chinese folk medicine, the leaves and bark of* M. glyptostroboides *are used as antimicrobic, analgesic, and anti-inflammatory drug for dermatic diseases. Thus far, the flavonoids, terpenoids, lignins, carotenoids, steroids, norlignans, and phenylpropanoids in the stems and leaves as well as essential oil in floral cones of* M. glyptostroboides* have been reported [10–14]. The total flavones extracts, essential oil, and some other chemical ingredients from this plant were proved to exhibit cardiovascular protective effect [15] and antibacterial, antioxidant, and cytotoxic activities [10, 11]. However, there is no report available in the literature on antioxidant properties of the bark of* M. glyptostroboides* and its active compounds. Hence, the present study was designed to evaluate the free radical scavenging capacity and antioxidant activity of the extracts from the bark of* M. glyptostroboides* and to isolate and identify the proanthocyanidin compounds in its active fractions.

## 2. Materials and Methods

### 2.1. Chemicals and Reagents

^1^H NMR, ^13^C NMR, and DEPT spectra were recorded at 500 MHz for ^1^H and at 125 MHz for ^13^C, with a Bruker DRX 500 instrument in DMSO-*d*_6_ solution. ESI-MS spectra were recorded on a Bruker Esquire 3000^plus^ mass spectrometer, using a negative ion probe with samples dissolved in methanol.

1,1-Diphenyl-2-picrylhydrazyl (DPPH), ferrozine, and Folin-Ciocalteu reagent were purchased from Sigma Aldrich Co (St. Louis, MO, USA). The reference standard Pycnogenol (Maritime Pine Extract, containing 673 mg proanthocyanidins per g material) was purchased from the United States Pharmacopeial Convention (Rockville, MD, USA). The reference standards gallic acid (purity ≥ 98%), rutin (purity ≥ 98%), proanthocyanidins (purity ≥ 95%), and ascorbic acid (purity ≥ 99%) were purchased from Internet Aladdin reagent database Inc. (Shanghai, China). The detection kits for protein content, total antioxidative capacity (T-AOC), superoxide anion free radical, hydroxyl free radical and malondialdehyde (MDA), aspartate aminotransferase (AST), alanine aminotransferase (ALT), superoxide dismutase (SOD), and reduced glutathione (GSH) were purchased from Nanjing Jiancheng Bioengineering Institute (Nanjing, Jiangsu, China). All other reagents were of analytical grade and made in China.

### 2.2. Plant Material

The barks of* M. glyptostroboides* were collected in the Hangzhou Xiaoshan, Zhejiang Province, China, in May 2010, and were identified according to the Flora of China by one of the authors (Dr. Xiaoyu Li). A voucher specimen (number 20100505) has been deposited at the Laboratory of Nature Drug, Institute of Materia Medica, Zhejiang Academy of Medical Sciences, China. The plant materials were dried under shade, pulverized, and stored at 4°C for further use.

### 2.3. Preparation of the Extracts and Fractions

The extracts and fractions were prepared from the barks of* M*.* glyptostroboides* under subdued light by water-ethanol extraction and D101 resin column chromatography. In detail, the pulverized bark of* M. glyptostroboides* (1.0 kg) was extracted three times with water, 60% or 95% ethanol under reflux for 2 h, respectively. The extract was filtered through Whatman filter paper. The filtrate was concentrated in a rotary evaporator under reduced pressure and then lyophilized to obtain water, 60% ethanol, and 95% ethanol extract, named as MGW (69 g), MGE60 (101 g), and MGE95 (70 g), respectively. The MGE60 (50 g) was then subjected to D101 resin column chromatography and eluted subsequently with water and 30% and 50% ethanol. Finally, the eluates were concentrated and lyophilized to yield water (MGEA, 7 g) and 30% (MGEB, 17 g) and 50% ethanol (MGEC, 2 g) fractions. All the extracts and fractions were stored at 4°C for subsequent analyses.

### 2.4. Isolation and Identification of Proanthocyanidins

The brown powder MGEB (100 g) prepared as the above method was subjected to Sephadex LH-20 column chromatography (EtOH/H_2_O 4 : 6 → 95 : 5 v/v, then acetone/H_2_O 2 : 8 → 6 : 4 v/v). The fraction eluted with EtOH/H_2_O (8 : 2, v/v) was separated by HPLC (MeOH/H_2_O) to give** 1 **(435 mg),** 2 **(13 mg),** 3 **(24 mg), and** 4 **(29 mg). The fractions eluted with EtOH/H_2_O (95 : 5, v/v) and acetone/H_2_O (2 : 8, v/v) were separated by HPLC (Agilent 1100 HPLC, column: Zorbax 300SB-C18 PrepHT, 21.2 × 250 mm, 7 *μ*m; mobile phase: MeOH : H_2_O = 65 : 35; flow rate: 8 mL/min) to give** 5 **(107 mg),** 6 **(16 mg),** 7 **(18 mg),** 8 **(89 mg),** 9 **(18 mg), and** 10 **(47 mg). All the compounds were identified by NMR and MS spectroscopy and by comparison with spectral literature data [16–22].

### 2.5. Total Phenolic, Flavonoid, and Proanthocyanidin Content Determination

The total phenolic and flavonoid contents of the sample were determined by the methods described in the Chinese Pharmacopoeia (Chinese Pharmacopoeia Committee, 2010). The total phenolic content was analyzed based on Folin-Ciocalteu colourimetric method, using gallic acid as a standard. The sample was dissolved in distilled water and diluted appropriately and then mixed with 1 mL Folin-Ciocalteu solution. The mixture was adjusted to 25 mL with 29% sodium carbonate and allowed to rest for 30 min in the dark. The absorbance (*A*) was measured at 760 nm, and the total phenolic content was expressed as gallic acid equivalents (mg GAE/g sample).

To determine the total flavonoid content, the sample was dissolved in 50% ethanol and mixed with 1 mL NaNO_2_ (5%). After standing for 6 min, 1 mL of 10% AlCl_3_ and 10 mL of NaOH (1.075 M) were added to the mixture. The mixture was adjusted to 25 mL with 50% ethanol and allowed to rest for 15 min. The absorbance (*A*) was measured at 510 nm. Rutin was used as a reference standard and the total flavonoid content was expressed as rutin equivalents (mg RE/g sample).

The total proanthocyanidin content of the sample was determined by the* n*-Butanol/HCl assay [23]. The sample was dissolved in methanol and diluted appropriately. 1 mL of sample solution was added to 6 mL of a 95% solution of* n*-Butanol/HCl (95 : 5 v/v) in stoppered test tubes followed by 0.25 mL of a solution of NH_4_Fe(SO_4_)_2_ *·*12H_2_O in 2 M HCl. The tubes were incubated for 40 min at 95°C. After being cooled in the dark, the absorbance (*A*) was measured at 550 nm. Proanthocyanidin standard was used as a reference and the total proanthocyanidin content was expressed as proanthocyanidin equivalents (mg PE/g sample). All these three content determinations were performed in triplicate.

### 2.6. Free Radical Scavenging Activity Determination

The DPPH radical scavenging activities were determined according to the method described by Yokozawa et al. [24] with some modification. Briefly, in 96-well flat-bottom plates, 100 *μ*L of 0.5 mM freshly prepared DPPH ethanol solution was added to 100 *μ*L of sample solution in 50% ethanol at different concentrations. The mixture was shaken vigorously and incubated for 30 min in the dark at room temperature. The absorbance of each reaction mixture was measured at 517 nm. Lower absorbance of the reaction mixture indicated higher free radical scavenging activity.

The scavenging effect on superoxide anion radical and hydroxyl radical were estimated using assay kits according to the manufacturer's instructions [25]. The superoxide anion radical and hydroxyl radical were generated by PMS-NADH system and Fenton reaction, respectively. The quantity of radical in system is determined using Griess reagent colorimetric method. Following manufactures instructions the reaction was conducted at 37°C in a water bath and the absorbance determined at 550 nm with the blank comprising reagents only.

For these three free radical scavenging assays, all the tests were performed in triplicate. Ascorbic acid was used as a standard and the antioxidant capacity was calculated as gram ascorbic acid equivalents per gram sample (g ascorbic acid eq./g).

### 2.7. Antioxidant Activity Determination

T-AOC was measured by ferric reducing antioxidant power assay (FRAP) [26] using assay kits according to the manufacturer's instructions. The tests were performed in triplicate. Ascorbic acid was used as a standard and the antioxidant capacity was calculated as gram ascorbic acid equivalents per gram sample (g ascorbic acid eq./g).

The lipid peroxidation inhibitory activity and ferrous ion (Fe^2+^) chelating activity were determined by the methods as our previously used [2]. Lipid peroxidation was induced by FeCl_2_-ascorbic acid in rat liver tissues. The level of lipid peroxidation was indicated by the amount of MDA assayed using detection kits by the thiobarbituric acid reaction (TBARS). Ferrous ion (Fe^2+^) chelating activity was indicated by inhibition of the formation of Fe^2+^-ferrozine complex. The rate of inhibition and half inhibitory concentration (IC_50_) value were calculated with the NDST software. Each test was performed in triplicate.

### 2.8. *In Vivo* Antioxidant Activity Determination

Six-week-old male ICR mice were purchased from Zhejiang Experimental Animal Center (Certificate number SCXK 2008-0033, Hangzhou, Zhejiang, China). Rodent laboratory chow and tap water were provided ad libitum and maintained under controlled conditions: temperature 24 ± 1°C, humidity 50 ± 10%, 12 h light/12 h dark cycle. All the procedures were in strict accordance with China's legislation on the use and care of laboratory animals and with the guidelines established by the Institute for Experimental Animals and were approved by the Committee for Animal Experiments.

The animals were randomly divided into five groups, each consisting of ten mice. The five groups are the normal group, the model control group, the two test groups, and the positive control group. Mice in the two test groups and the positive control group were treated with MGEB at a dose of 20, 80 mg/kg, or silymarin at a dose of 20 mg/kg body weight for seven consecutive days (p.o., once per day), while mice in normal group and model group were treated with physiological saline. The mice were injected with CCl_4_ (10 mL/kg i.p. of 0.125% CCl_4_ solution in olive oil) one hour after the sixth administration, except for the normal group, which was given only olive oil injection. The animals were sacrificed 24 h after the CCl_4_ treatment. Serum was collected for ALT and AST determination. Liver was collected for SOD, MDA, T-AOC, and GSH investigations and histopathologic analysis.

The activities of ALT, AST, SOD, MDA, T-AOC, and GSH were determined by assay kits according to the manufacturer's instructions. Pieces of liver tissues were stained with hematoxylin-eosin (HE) and examined under light microscope (Olympus, Japan) for general histopathology examination.

### 2.9. Statistical Analysis

The data were expressed as mean ± standard deviation (SD) and examined for their statistical significance of difference with ANOVA and the Standard's* t*-test. *P* values of less than 0.05 were considered to be statistically significant. Correlation analysis were carried out using the correlation and regression program in Microsoft Office Excel to study the relationship between free radical scavenging, antioxidant activities, and total phenolic, flavonoid, and proanthocyanidin content.

## 3. Results

### 3.1. Total Phenolic, Flavonoid, and Proanthocyanidin Contents

The contents of total phenolics, flavonoids, and proanthocyanidins in each extract and fraction were determined by Folin-Ciocalteu colourimetric, aluminium colourimetric, and* n*-Butanol/HCl method, respectively. As shown in Table 1, among three extracts, the 95% ethanol extract (MGE95) had the highest contents of total phenolics and proanthocyanidins. The total phenolic, flavonoid, and proanthocyanidin contents in MGEB, obtained by 60% ethanol extraction and 30% ethanol elution with D101 resin column chromatograph, were significantly higher than those of crude extracts and other two fractions. Meanwhile, the yield of MGEB was also higher than that of other two fractions. Furthermore, MGEB are close to Pycnogenol in the total proanthocyanidin contents, being 627.5 ± 8.7 mg PE/g and 650–750 mg PE/g, respectively.

### 3.2. Free Radical Scavenging Capacity

**Figure 1:**
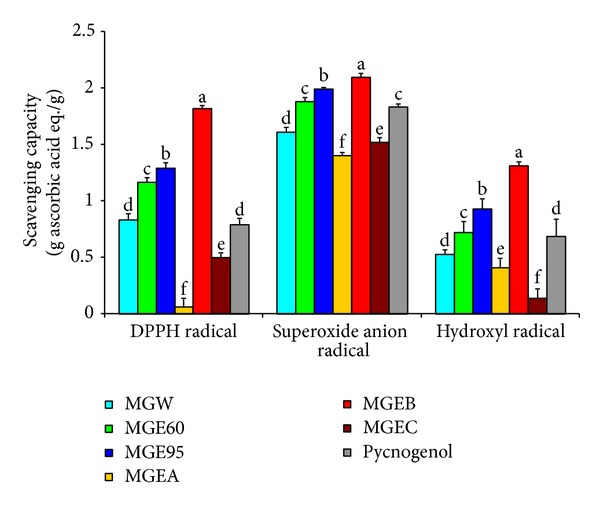
Free radical scavenging capacity of the extracts and fractions from the bark of* M. glyptostroboides.* Values are mean ± standard deviation of three replicate analyses. Means with different letters (a–f) within the same column differed significantly (*P* < 0.05) and are arranged in the following scavenging capacity descending order: a > b> c > d > e > f.

All the extracts and fractions dose dependently showed DPPH, superoxide anion, and hydroxyl radical scavenging capacities at the concentrations of 2~200 *μ*g/mL (data no shown). As shown in Figure 1, the DPPH radical scavenging capacity was arranged in the following descending order: MGEB > MGE95 > MGE60 > MGW > MGEC > MGEA (*P* < 0.05). Among them, MGEB, MGE95, MGE60, and MGW showed higher scavenging capacity than Pycnogenol. Moreover, the DPPH radical scavenging capacity of MGEB, MGE95, and MGE60 were higher than that of ascorbic acid (values > 1).

MGEB also possessed the strongest superoxide anion and hydroxyl radical scavenging capacity. Moreover, the arrangement order of superoxide anion radical scavenging capacity of extracts and fractions is consistent with the findings in DPPH radical scavenging capacity. All of the samples including Pycnogenol showed higher superoxide anion radical scavenging capacity than that of ascorbic acid (values > 1), while only MGEB showed higher hydroxyl radical scavenging capacity than that of ascorbic acid. We also found that both superoxide anion radical and hydroxyl radical scavenging capacities of MGEB and MGE95 were higher than that of Pycnogenol (*P* < 0.05).

### 3.3. Antioxidant Activity

**Figure 2:**
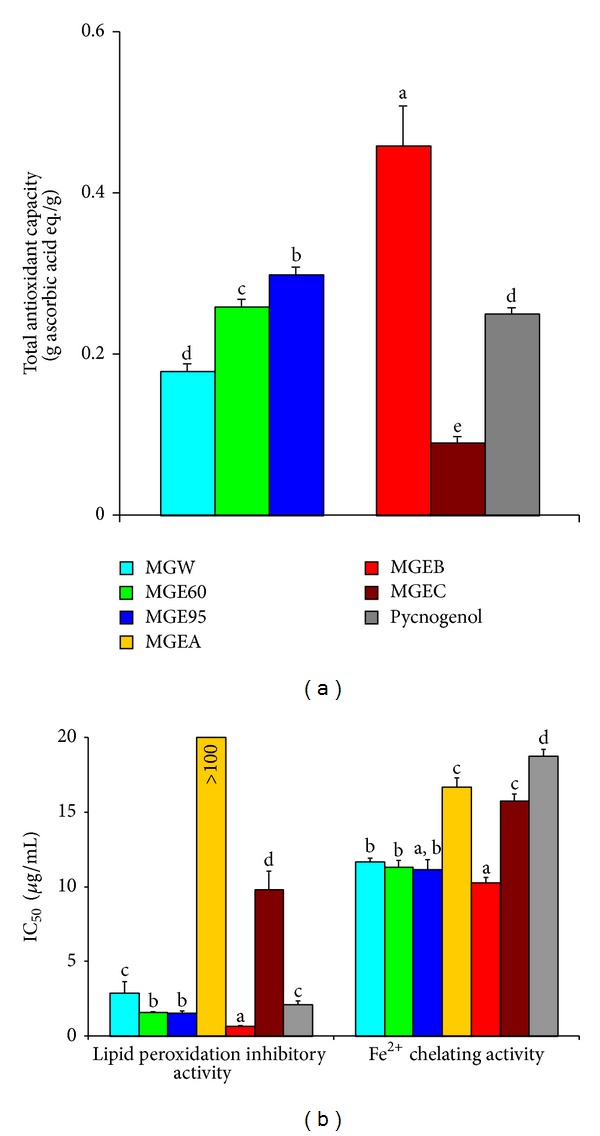
Antioxidant activity of the extracts and fractions from the bark of* M. glyptostroboides.* Values are mean ± standard deviation of three replicate analyses. Means with different letters (a–f) within the same column differed significantly (*P* < 0.05) and are arranged in the following antioxidant activity descending order: a > b> c > d > e > f.

T-AOC of the extracts and fractions from the bark of* M. glyptostroboides *were measured by FRAP assay [26]. As shown in Figure 2(a), the T-AOC was arranged in the following descending order: MGEB > MGE95 > MGE60 > MGEW > MGEC > MGEA (*P* < 0.05). Though the T-AOC of the extracts and fraction samples were not higher than that of ascorbic acid (values < 1), MGEB and MGE95 showed higher antioxidant activities than Pycnogenol (*P* < 0.05).

The lipid peroxidation inhibitory activity was shown in Figure 2(b). All the extracts and fractions exhibited significant lipid peroxidation inhibitory activities. Moreover, MGEB, MGE95, and MGE60 showed higher activities than Pycnogenol (*P* < 0.05).

All the extracts and fractions exhibited moderate Fe^2+^ chelating activities (Figure 2(b)). The IC_50_ values of these extracts were higher than those of the positive standard EDTA (IC_50_: 4.24 ± 0.08 *μ*g/mL), but lower than those of Pycnogenol (*P* < 0.05). Being consistent with the findings above, MGEB possessed the strongest Fe^2+^ chelating activity.

### 3.4. Relationship between Free Radical Scavenging, Antioxidant Activities, and the Content of Total Phenolics, Flavonoids, and Proanthocyanidins

The relationship between free radical scavenging, antioxidant activities, and the content of total phenolics, flavonoids, and proanthocyanidins was further analyzed by the correlation and regression program, and the results were shown in Table 2. The correlation coefficient (*R*) between the total contents and the free radical scavenging activities or total antioxidant capacity was found to be significant. However, the poor correlation coefficient was observed between the total contents and the lipid peroxidation inhibitory activity or Fe^2+^ chelating activity. These results were similar to the studies conducted by Liu et al. [27] in emblica fruit. In addition, the correlation coefficient between the total proanthocyanidin contents and the free radical scavenging or antioxidant activities was shown to be most significant for all the cases, especially in hydroxyl radical and superoxide anion radical.

### 3.5. *In Vivo* Antioxidant Activity of MGEB

**Figure 3:**
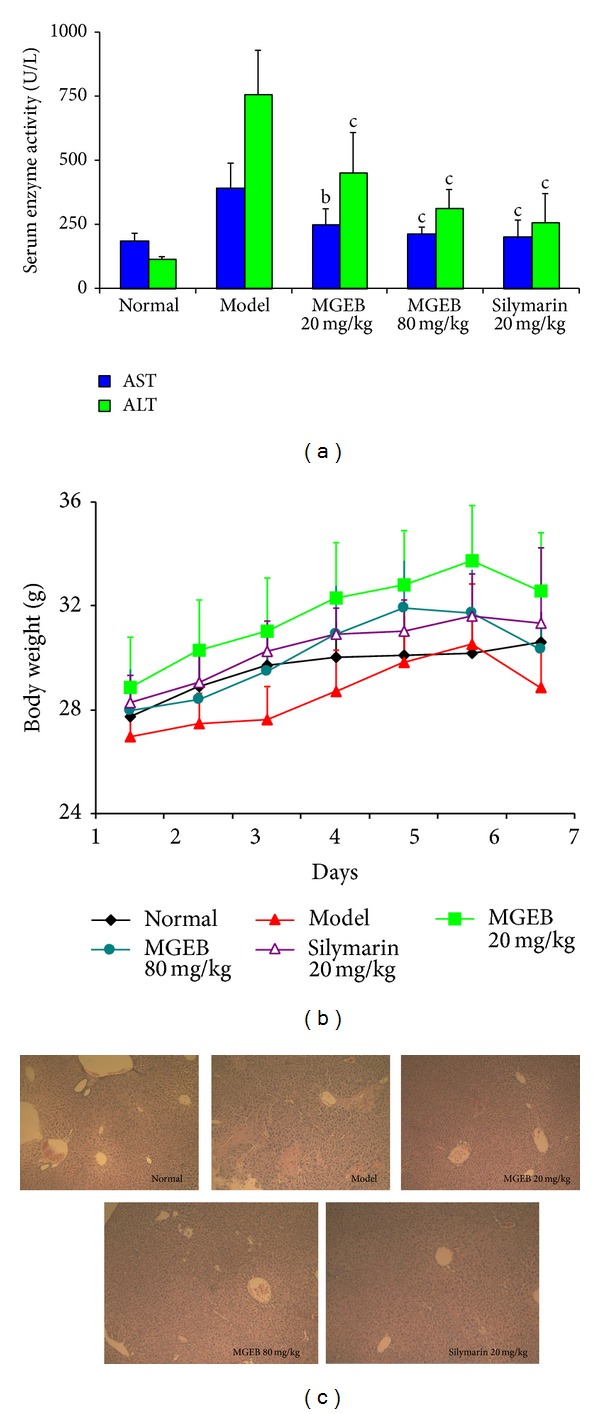
MGEB protect against CCl_4_ induced acute liver injury of mice. (a) Serum hepatic enzyme activity of ALT and AST. The data were expressed as mean ± standard deviation (*n* = 10); significant differences with model group were designated as ^b^ *P* < 0.01, ^c^ *P* < 0.001. (b) Body weights of the mice were measured every day. Data represent the mean ± standard deviation (*n* = 10). (c) Slices of liver were stained with hematoxylin and eosin for histopathological analysis. Representative histomicrographs of liver sections of each group.

**Figure 4:**
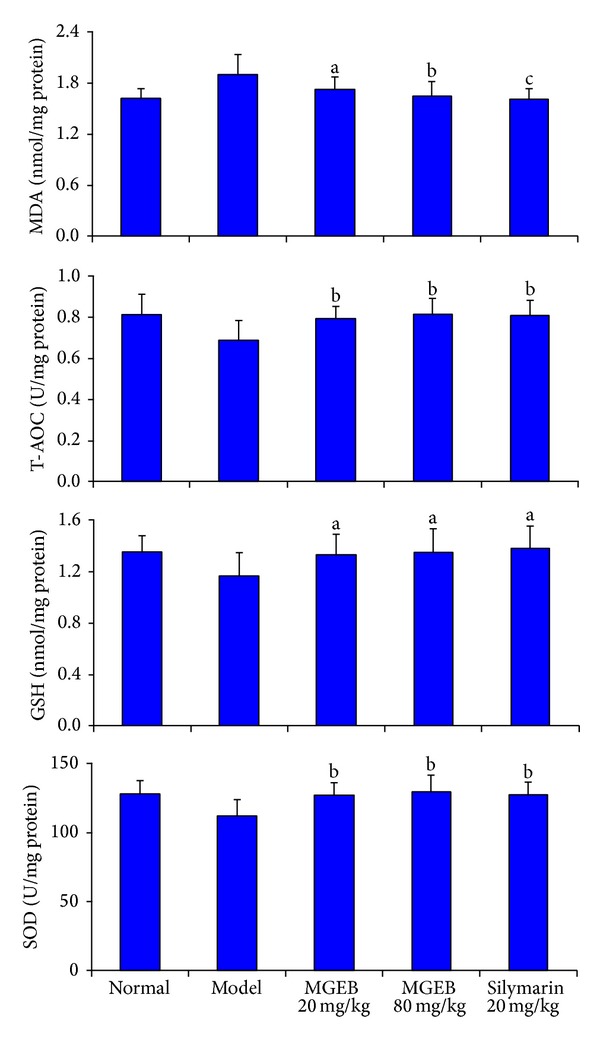
MGEB protect against CCl_4_ induced oxidative stress in liver of mice. The data were expressed as mean ± standard deviation (*n* = 10); significant differences with model group were designated as ^a^ *P* < 0.05, ^b^ *P* < 0.01, and ^c^ *P* < 0.001.

The* in vivo* antioxidant activity of MGEB was evaluated for CCl_4_ induced acute liver injury in mice. CCl_4_ administration at day 6 resulted in a slight decrease of body weight (Figure 3(b)) and significant elevation of serum AST and ALT activity (Figure 3(a)). Histopathological examination of livers challenged with CCl_4_ showed vacuole formation, inflammatory infiltration, and widespread necrotic cells (Figure 3(c)). CCl_4_ also significantly induced oxidative stress in the liver (Figure 4). CCl_4_ caused a significant increase of MDA level and decrease of T-AOC, SOD, and GSH levels in the liver. Oral administration of MGEB at the doses of 20, 80 mg/kg or silymarin at the dose of 20 mg/kg body weight prior to CCl_4_ treatment significantly restored the activities of AST and ALT. The histological observations basically supported these results. MGEB and silymarin were able to prevent the development of histopathological changes, which exhibited areas of normal liver architecture and patches of inflammatory infiltration and necrotic hepatocytes. In addition, MGEB and silymarin significantly reversed the CCl_4_ induced MDA elevation and T-AOC, SOD, and GSH descent. Moreover, no signs of toxicity were observed in the MGEB-treated mice on the basis of body weight and microscopic examination of individual organs. These findings indicated that MGEB could significantly protect against CCl_4_ induced acute liver injury through inhibition of lipid peroxidation and the increase of antioxidant activity.

### 3.6. Structural Elucidation of Proanthocyanidin Composition from MGEB

**Figure 5:**
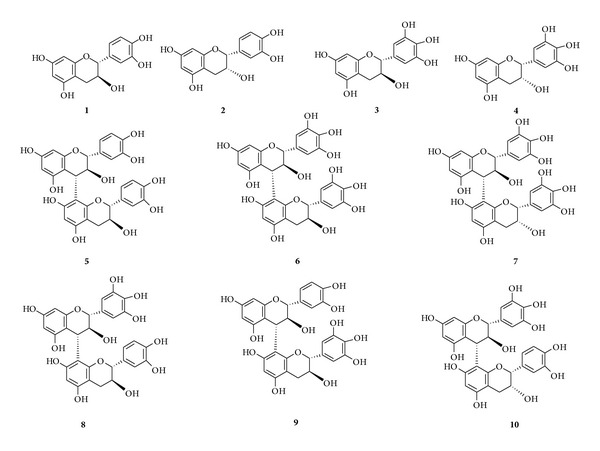
Proanthocyanidins from the bark of* M. glyptostroboides*.

Ten proanthocyanidins were isolated and identified from MGEB; the structure elucidations of them were identified by spectral techniques as ES-MS, ^1^H and ^13^C NMR (Figure 5). Previous phytochemical investigations have reported the presence of compounds 1–4 from the branches and stems of* M. glyptostroboides* [14]. Compounds 5–10 are reported for the first time from this plant.

#### 3.6.1. Catechin ****(1)****

ESI-MS gave an [M-H]^−^ at* m/z*: 289. ^1^H NMR (500 MHz, DMSO-*d*_6_):  **δ** 6.73 (1H, d,* J* = 2.0 Hz, H-2′), 6.70 (1H, d,* J* = 8.0 Hz, H-6′), 6.61 (1H, dd,* J* = 2.0, 8.0 Hz, H-5′), 5.90 (1H, d,* J* = 2.0 Hz, H-8), 5.69 (1H, d,* J* = 2.0 Hz, H-6), 4.49 (1H, d,* J* = 7.5 Hz, H-2), 3.82 (1H, m, H-3), 2.64 (1H, m, H-4*α*), 2.33 (1H, m, H-4*β*). ^13^C NMR (125 MHz, DMSO-*d*_6_): **δ** 156.40 (C-7), 156.15 (C-5), 155.34 (C-8a), 144.82 (C-3′, 4′), 130.58 (C-1′), 118.41 (C-6′), 115.04 (C-5′), 114.49 (C-2′), 99.04 (C-4a), 95.05 (C-6), 93.79 (C-8), 80.98 (C-2), 66.28 (C-3), 27.86 (C-4).

#### 3.6.2. Epicatechin ****(2)****

ESI-MS gave an [M-H]^−^ at* m/z*: 289. ^1^H NMR (500 MHz, DMSO-*d*_6_): **δ** 6.89 (1H, br s, H-2′), 6.68 (1H, d,* J* = 8.5 Hz, H-6′), 6.66 (1H, d,* J* = 8.5 Hz, H-5′), 5.89 (1H, d,* J* = 1.5 Hz, H-6), 5.72 (1H, d,* J* = 2.0 Hz, H-8), 4.73 (1H, s, H-2), 4.00 (1H, d,* J* = 2.5 Hz, H-3), 2.70 (1H, dd,* J* = 4.5, 16.5 Hz, H-4*α*), 2.46 (1H, dd,* J* = 3.0, 16.5 Hz, H-4*β*). ^13^C NMR (125 MHz, DMSO-*d*_6_): **δ** 156.48 (C-7), 156.20 (C-5), 155.73 (C-8a), 144.46 (C-4′), 144.40 (C-3′), 130.58 (C-1′), 117.92 (C-6′), 114.87 (C-5′), 114.73 (C-2′), 98.48 (C-4a), 95.06 (C-6), 94.07 (C-8), 78.02 (C-2), 64.88 (C-3), 28.14 (C-4).

#### 3.6.3. Gallocatechin ****(3)****

ESI-MS gave an [M-H]^−^ at* m/z*: 305. ^1^H NMR (500 MHz, DMSO-*d*_6_): **δ** 6.25 (2H, s, H-2′, 6′), 5.88 (1H, d,* J* = 2.0 Hz, H-8), 5.69 (1H, d,* J* = 2.0 Hz, H-6), 4.44 (1H, d,* J* = 7.0 Hz, H-2′), 3.80 (1H, m, H-3), 2.63 (1H, dd,* J* = 5.5, 16.5 Hz, H-4*α*), 2.37 (1H, dd,* J* = 7.5, 16.5 Hz, H-4*β*). ^13^C NMR (125 MHz, DMSO-*d*_6_): **δ** 156.36 (C-7), 156.11 (C-5), 155.23 (C-8a), 145.57 (C-3′, 5′), 132.44 (C-4′), 129.8 (C-1′), 105.95 (C-2′, 6′), 98.95 (C-4a), 95.05 (C-6), 93.82 (C-8), 80.98 (C-2), 66.25 (C-3), 27.33 (C-4).

#### 3.6.4. Epigallocatechin ****(4)****

ESI-MS gave an [M-H]^−^ at* m/z*: 305. ^1^H NMR (500 MHz, DMSO-*d*_6_): **δ** 6.37 (2H, s, H-2′, 6′), 5.89 (1H, d,* J* = 2.0 Hz, H-8), 5.71 (1H, d,* J* = 2.0 Hz, H-6), 4.66 (1H, s, H-2), 4.13 (1H, m, H-3), 2.69 (1H, dd,* J* = 4.5, 16.5 Hz, H-4*α*), 2.37 (1H, dd,* J* = 3.0, 16.5 Hz, H-4*β*). ^13^C NMR (125 MHz, DMSO-*d*_6_): **δ** 156.46 (C-7), 156.18 (C-5), 155.71 (C-8a), 146.33 (C-3′, 5′), 132.09 (C-4′), 129.69 (C-1′), 106.03 (C-2′, 6′), 98.54 (C-4a), 95.03 (C-6), 94.05 (C-8), 78.09 (C-2), 64.96 (C-3), 28.09 (C-4).

#### 3.6.5. Catechin (4*α* → 8) Catechin ****(5)****

ESI-MS gave an [M-H]^−^ at* m/z*: 577.8. ^1^H NMR (500 MHz, DMSO-*d*_6_): Upper (catechin) unit: **δ** 6.84 (1H, d,* J* = 2.0 Hz, H-2′), 6.76 (1H, dd,* J* = 8.0, 2.0 Hz, H-6′), 6.70 (1H, d,* J* = 8.0 Hz, H-5′), 5.67 (1H, d,* J* = 2.5 Hz, H-8), 5.61 (1H, d,* J* = 2.5 Hz, H-6), 4.26 (1H, d,* J* = 7.5 Hz, H-4), 4.21 (1H, m, H-3), 4.17 (1H, d,* J* = 9.5 Hz, H-2). Lower (catechin) unit: **δ** 6.82 (1H, d,* J* = 2.0 Hz, H-2′), 6.65 (1H, dd,* J* = 8.0, 2.0 Hz, H-6′), 6.58 (1H, d,* J* = 8.0 Hz, H-5′), 5.85 (1H, s, H-6), 4.72 (1H, d,* J* = 7.5 Hz, H-2), 3.86 (1H, m, H-3), 2.81 (1H, dd,* J* = 16.5, 6.0 Hz, H-4*α*), 2.41 (1H, dd,* J* = 16.5, 8.5 Hz, H-4*β*). ^13^C NMR (125 MHz, DMSO-*d*_6_): Upper (catechin) unit: **δ**  154.53 (C-7), 153.48 (C-5), 153.29 (C-8a), 144.83 (C-3′), 144.68 (C-4′), 130.81 (C-1′), 119.39 (C-6′), 115.15 (C-5′), 114.79 (C-2′), 106.16 (C-4a), 95.93 (C-8), 94.14 (C-6), 82.51 (C-2), 71.05 (C-3), 37.29 (C-4). Lower (catechin) unit: **δ**  157.34 (C-7), 155.99 (C-5), 155.50 (C-8a), 144.72 (C-3′), 144.56 (C-4′), 130.96 (C-1′), 118.80 (C-6′), 115.20 (C-5′), 114.92 (C-2′), 108.40 (C-8), 98.95 (C-4a), 95.89 (C-6), 81.42 (C-2), 66.68 (C-3), 29.49 (C-4).

#### 3.6.6. Gallocatechin (4*α* → 8) Gallocatechin ****(6)****

ESI-MS gave an [M-H]^−^ at* m/z*: 609.8. ^1^H NMR (500 MHz, DMSO-*d*_6_): Upper (gallocatechin) unit: **δ**  6.57 (1H, s, H-2′), 6.57 (1H, s, H-6′), 5.89 (1H, d,* J* = 2.5 Hz, H-8), 5.85 (1H, d,* J* = 2.5 Hz, H-6), 4.56 (1H, d,* J* = 8.0 Hz, H-4), 4.44 (1H, d,* J* = 8.0 Hz, H-3), 4.23 (1H, d,* J* = 9.5 Hz, H-2). Lower (gallocatechin) unit: **δ**  6.58 (1H, s, H-2′), 6.58 (1H, s, H-6′), 6.04 (1H, s, H-6), 4.67 (1H, d,* J* = 7.0 Hz, H-2), 4.11 (1H, d,* J* = 6.5 Hz H-3), 2.79 (1H, dd,* J* = 16.0, 6.0 Hz, H-4*α*), 2.40 (1H, dd,* J* = 16.5, 8.5 Hz, H-4*β*). ^13^C NMR (125 MHz, DMSO-*d*_6_): Upper (gallocatechin) unit: **δ**  157.33 (C-7), 155.98 (C-5), 155.51 (C-8a), 145.42 (C-3′, C-5′), 132.54 (C-4′), 130.05 (C-1′), 107.00 (C-2′, C-6′), 106.09 (C-4a), 95.88 (C-8), 94.11 (C-6), 82.76 (C-2), 71.03 (C-3), 37.07 (C-4). Lower (gallocatechin) unit: **δ**  154.57 (C-7), 153.47 (C-5), 153.32 (C-8a), 145.42 (C-3′, C-5′), 132.54 (C-4′), 129.87 (C-1′), 108.35 (C-8), 106.74 (C-2′, C-6′), 99.00 (C-4a), 95.88 (C-6), 81.78 (C-2), 66.65 (C-3), 29.47 (C-4).

#### 3.6.7. Gallocatechin (4*α* → 8) Epigallocatechin ****(7)****

ESI-MS gave an [M-H]^−^ at* m/z*: 609.8. ^1^H NMR (500 MHz, DMSO-*d*_6_): Upper (gallocatechin) unit: **δ**  6.59 (1H, s, H-2′, H-6′), 5.88 (1H, d,* J* = 2.5 Hz, H-8), 5.86 (1H, d,* J* = 2.5 Hz, H-6), 4.68 (1H, d,* J* =7.5 Hz, H-4), 4.51 (1H, d,* J* = 8.0 Hz, H-3), 4.38 (1H, d,* J* = 9.5 Hz, H-2). Lower (epigallocatechin) unit:**δ** 6.71 (1H, s, H-2′, H-6′), 6.04 (1H, s, H-6), 4.93 (1H, d,* J* = 9.5 Hz, H-2), 4.25 (1H, br s, H-3), 2.83 (1H, dd,* J* = 17.0, 2.5 Hz, H-4*α*), 2.74 (1H, dd,* J* = 16.5, 8.5 Hz, H-4*β*). ^13^C NMR (125 MHz, DMSO-*d*_6_): Upper (gallocatechin) unit: **δ**  157.34 (C-7), 156.00 (C-5), 155.51 (C-8a), 145.42 (C-3′, C-5′), 132.54 (C-4′), 129.97 (C-1′), 107.04 (C-2′, C-6′), 106.05 (C-4a), 95.91 (C-8), 94.15 (C-6), 82.53 (C-2), 71.24 (C-3), 37.24 (C-4). Lower (epigallocatechin) unit: **δ**  154.63 (C-7), 153.84 (C-5), 153.46 (C-8a), 145.29 (C-3′, C-5′), 132.05 (C-4′), 130.11 (C-1′), 108.24 (C-8), 106.16 (C-2′, C-6′), 97. 91 (C-4a), 95.77 (C-6), 78.39 (C-2), 65.14 (C-3), 29.18 (C-4).

#### 3.6.8. Gallocatechin (4*α* → 8) Catechin ****(8)****

ESI-MS gave an [M-H]^−^ at* m/z*: 593.9. ^1^H NMR (500 MHz, DMSO-*d*_6_): Upper (gallocatechin) unit: **δ**  6.57 (1H, s, H-2′, H-6′), 5.95 (1H, d,* J* = 2.0 Hz, H-8), 5.80 (1H, d,* J* = 2.5 Hz, H-6), 4.65 (1H, d,* J* = 7.0 Hz, H-4), 4.54 (1H, d,* J* = 9.0 Hz, H-3), 4.38 (1H, d,* J* = 9.5 Hz, H-2). Lower (catechin) unit: **δ**  7.03 (1H, s, H-2′), 6.90 (1H, dd,* J* = 8.5, 2.0 Hz, H-6′), 6.79 (1H, d,* J* = 8.0 Hz, H-5′), 6.05 (1H, s, H-6), 4.75 (1H, d,* J* = 7.5 Hz, H-2), 4.09 (1H, m, H-3), 2.92 (1H, dd,* J* = 16.5, 5.5 Hz, H-4*α*), 2.65 (1H, dd,* J* = 16.5, 8.5 Hz, H-4*β*). ^13^C NMR (125 MHz, DMSO-*d*_6_): Upper (gallocatechin) unit: **δ**  157.34 (C-7), 155.98 (C-5), 155.49 (C-8a), 145.40 (C-3′, C-5′), 132.54 (C-4′), 130.04 (C-1′), 107.04 (C-2′, C-6′), 106.13 (C-4a), 95.89 (C-8), 94.13 (C-6), 82.80 (C-2), 71.06 (C-3), 37.09 (C-4). Lower (catechin) unit: **δ**  154.52 (C-7), 153.48 (C-5), 153.28 (C-8a), 144.71 (C-3′), 144.56 (C-4′), 130.81 (C-1′), 118.80 (C-6′), 115.12 (C-5′), 114.93 (C-2′), 108.41 (C-8), 98.95 (C-4a), 95.89 (C-6), 81.42 (C-2), 66.70 (C-3), 29.47 (C-4).

#### 3.6.9. Catechin (4*α* → 8) Gallocatechin ****(9)****

ESI-MS gave an [M-H]^−^ at* m/z*: 593.9. ^1^H NMR (500 MHz, DMSO-*d*_6_): Upper (catechin) unit: **δ**  7.01 (1H, s, H-2′), 6.86 (1H, dd,* J* = 8.0, 2.0 Hz, H-6′), 6.82 (1H, d,* J* = 8.0 Hz, H-5′), 5.87 (1H, s, H-8), 5.86 (1H, s, H-6), 4.45 (1H, d,* J* = 7.5 Hz, H-4), 4.41 (1H, dd,* J* = 9.5, 7.5 Hz, H-3), 4.27 (1H, d,* J* = 9.5 Hz, H-2). Lower (gallocatechin) unit: **δ**  6.58 (1H, s, H-2′, H-6′), 6.05 (1H, s, H-6), 4.66 (1H, d,* J* = 7.5 Hz, H-2), 4.08 (1H, m, H-3), 2.92 (1H, dd,* J* = 16.0, 5.5 Hz, H-4*α*), 2.64 (1H, dd,* J* = 16.5, 8.5 Hz, H-4*β*). ^13^C NMR (125 MHz, DMSO-*d*_6_): Upper (catechin) unit: **δ**  154.59 (C-7), 153.48 (C-5), 153.32 (C-8a), 144.84 (C-3′), 144.68 (C-4′), 129.86 (C-1′), 119.37 (C-6′), 115.17 (C-5′), 114.80 (C-2′), 106.10 (C-4a), 94.92 (C-8), 94.13 (C-6), 82.48 (C-2), 71.01 (C-3), 37.21 (C-4). Lower (gallocatechin) unit: **δ**  157.31 (C-7), 155.99 (C-5), 155.51 (C-8a), 145.42 (C-3′, C-5′), 132.55 (C-4′), 130.95 (C-1′), 108.33 (C-8), 106.76 (C-2′, C-6′), 99.01 (C-4a), 95.92 (C-6), 81.78 (C-2), 66.63 (C-3), 29.48 (C-4).

### 3.7. Gallocatechin (4*α* → 8) Epicatechin ****(10)****

ESI-MS gave an [M-H]^−^ at* m/z*: 593.9. ^1^H NMR (500 MHz, DMSO-*d*_6_): Upper (gallocatechin) unit: **δ**  6.49 (1H, s, H-2′, H-6′), 5.85 (1H, d,* J* = 2.0 Hz, H-8), 5.67 (1H, d,* J* = 2.5 Hz, H-6), 4.48 (1H, d,* J* = 7.0 Hz, H-4), 4.41 (1H, d,* J* = 9.0 Hz, H-3), 4.25 (1H, d,* J* = 9.5 Hz, H-2). Lower (epicatechin) unit: **δ**  6.97 (1H, s, H-2′), 6.83 (1H, dd,* J* = 8.5, 2.0 Hz, H-6′), 6.67 (1H, d,* J* = 8.0 Hz, H-5′), 5.86 (1H, s, H-6), 4.73 (1H, br s, H-2), 4.11 (1H, m, H-3), 2.92 (1H, dd,* J* = 16.5, 4.5 Hz, H-4*α*), 2.53 (1H, dd,* J* = 16.5, 2.5 Hz, H-4*β*). ^13^C NMR (125 MHz, DMSO-*d*_6_): Upper (gallocatechin) unit: **δ**  155.98 (C-7), 155.51 (C-5), 155.50 (C-8a), 145.41 (C-3′, C-5′), 132.53 (C-4′), 131.02 (C-1′), 107.06 (C-4a), 106.17 (C-2′, C-6′), 95.91 (C-6), 94.18 (C-8), 82.55 (C-2), 71.27 (C-3), 37.27 (C-4). Lower (epicatechin) unit: **δ**  157.38 (C-7), 157.33 (C-5), 156.01 (C-8a), 144.68 (C-3′), 144.84 (C-4′), 132.06 (C-1′), 119.38 (C-6′), 115.22 (C-5′), 114.74 (C-2′), 108.23 (C-8), 97.91 (C-4a), 95.95 (C-6), 78.40 (C-2), 65.12 (C-3), 29.20 (C-4).

## 4. Discussion

It has been recognized that the free radical scavenging capacity and antioxidant activity of majority plant extracts are associated with their phenolic, flavonoid, or proanthocyanidin contents [28–30]. To obtain effective free radical scavenging and antioxidant materials from the bark of* M. glyptostroboides*, in present study, gradient water-ethanol (water, 60% or 95% ethanol) were first used as an extraction solvent to get the crude extracts, and the contents of total phenolic, flavonoid, and proanthocyanidin in each extract were determined. As shown in Table 1, among all three extracts, although the 95% ethanol extract (MGE95) had the highest contents of total phenolics and proanthocyanidins, the 60% ethanol extract (MGE60) obtained the highest extraction yield. The yields of total phenolics, flavonoids, and proanthocyanidins were higher in 60% ethanol extract (MGE60) than those in 95% ethanol extract (MGE95). Subsequently, the 60% ethanol extract (MGE60) was selected for further study.

In this study, the 60% ethanol extract was then subjected to D101 resin column chromatography and eluted with a water-ethanol step gradient to afford three fractions, MGEA (water fraction), MGEB (30% ethanol fraction), and MGEC (50% ethanol fraction). As shown in Table 1, the total phenolic, flavonoid, and proanthocyanidin contents in MGEB were significantly higher than those of its parent extracts and other two fractions, and its yield was also higher than that of other two fractions. These data suggest that resin column chromatography is suitable for enrichment of the phenolics, flavonoids, and proanthocyanidins from extracts of the bark of* M. glyptostroboides.*

DPPH is a nitrogen-centered stable free radical, and their color changes from violet to yellow when it is reduced by the process of either hydrogen or electron donation. Among the oxygen radicals, hydroxyl radical is the most reactive in biological systems and has been implicated as a highly damaging species in free radical pathology, which is capable of damaging almost every molecule found in living cells. This radical has the capacity to join nucleotides in DNA and cause strand breakage which contributes to carcinogenesis, mutagenesis, and cytotoxicity [2]. Superoxide anion is a weak oxidant; however, it plays important roles in the formation of powerful and dangerous ROS such as hydrogen peroxide, hydroxyl radical, and singlet oxygen, which induce oxidative damage in lipids, proteins, and DNA [29, 31]. Therefore, these three free radicals were used for comparison of the free radical scavenging capacity of the extracts and fractions from the bark of* M. glyptostroboides*. As shown in Figure 1, all the extracts and fractions seem to be good scavengers of free radicals, and MGEB possessed strongest capacity.

Lipid peroxidation in biological systems has long been thought to be a toxicological phenomenon that can lead to various pathological consequences. Fe^2+^-ascorbic acid mixture is well known to stimulate lipid peroxidation in rat liver* in vivo* and in microsome and mitochondria of rat liver* in vitro*. Metal chelating capacity is an important antioxidant property since it reduced the concentration of the catalysing transition metal in lipid peroxidation. Fe^2+^ is the most powerful prooxidant among the various species of catalysing transition metal ions [2]. To better characterize the antioxidant potentials, in this study, the extracts and fractions from the bark of* M. glyptostroboides *were assessed for the T-AOC, the lipid peroxidation inhibitory activity, and Fe^2+^ chelating activity. Consistent with the findings in free radical scavenging capacity, all the extracts and fractions exhibited a significant antioxidant activity, and MGEB possessed the strongest capacity (Figure 2).

Previously, we and others have reported that extracts or compounds with chelating activity are believed to inhibit lipid peroxidation by stabilizing transition metals chelating agents [2, 32]. However, in this study, the IC_50_ values of all the extracts and fractions except the fraction MGEA in Fe^2+^ chelating activity were much higher than those in lipid peroxidation inhibitory activity. These results means that although these extracts and fractions possess marked capacities for iron binding, their lipid peroxidation inhibitory activity may not be directly related to the iron binding capacity.

To further evaluate the antioxidant activity of the bark of* M. glyptostroboides in vivo*, the effect of MGEB on CCl_4_ induced oxidative stress was assessed. CCl_4_ is metabolized by hepatic microsomal cytochrome (CYP)2E1, CYP2B1, or CYP2B2 to form a reactive trichloromethyl (CCl_3_^∙^) radical. This radical can bind to cellular molecules (nucleic acid, protein, and lipid), impairing crucial cellular processes such as lipid metabolism, damage antioxidant enzymes such as SOD, and depletion on the levels of GSH which have a key role in coordinating innate antioxidant defense mechanisms and eventually leading to lipid peroxidation, cell necrosis, and the leakage of the marker enzymes such as AST and ALT into serum [33, 34]. In this study, pretreatment with MGEB significantly attenuated CCl_4_-induced liver damage in mice, evidenced by decreased serum enzyme activities of ALT and AST, which was also supported by the histopathological examination of the mice liver (Figure 3). MGEB also showed a significant protective effect against CCl_4_ induced hepatic MDA elevation and depletion of T-AOC, SOD, and GSH content (Figure 4). These findings indicated that MGEB has potent free radical scavenging and antioxidant activities both* in vitro* and* in vivo*.

Proanthocyanidins are polymeric flavan-3-ols which can serve as electron donor to terminate the radical chain reaction. Their antioxidant activity is dependant not only on the concentration, but also on the structure of individual compounds (such as the number of phenolic hydroxyl groups and their position), the degree of polymerization, and the interaction between the antioxidants [30]. As strong antioxidant, Pycnogenol is utilized for various diseases. Between 65% and 75% of Pycnogenol is proanthocyanidins, mainly comprising of catechin and epicatechin subunits with varying chain lengths [8]. The relationship analysis between free radical scavenging, antioxidant activities, and the content of total phenolics, flavonoids, and proanthocyanidins (Table 2) showed that proanthocyanidin compounds were major contributors to the free radical scavenging capacity and antioxidant activity of the extracts and fractions from the bark of* M. glyptostroboides*. To identify the active compounds, MGEB, which possessed the highest proanthocyanidins content and the highest free radical scavenging and antioxidant activities, was subjected to Sephadex LH-20 column chromatograph. The proanthocyanidins in* M. glyptostroboides* contain not only catechin and epicatechin, but also gallocatechin and epigallocatechin subunits (Figure 5), which are highly heterogeneous from Pycnogenol. It is reported that gallocatechins with a 3′,4′,5′-trihydroxyphenyl B-ring are more efficient free radical scavengers than catechins [28]. This may be the reason why several extracts and fractions such as MGEB and MGE95 are with lower total proanthocyanidin content, but exhibited stronger free radical scavenging capacity and antioxidant activity than Pycnogenol.

## 5. Conclusion

As far as we know, this study is the first to report the free radical scavenging capacity, antioxidant activity, and proanthocyanidin composition of the bark of* M. glyptostroboides*. From the present findings, it was concluded that the extracts and fractions from the bark of* M. glyptostroboides* have DPPH radical, superoxide anion radical, and hydroxyl radical scavenging capacity, ferric reducing ability, lipid peroxidation inhibitory activity, and metal ions chelating capacity. It also provided evidence that these activities were mainly related to their proanthocyanidin content. In addition, the data indicated that the fraction MGEB, which was obtained by 60% ethanol extraction and further separation with D101 resin column chromatograph, possessed the highest proanthocyanidin content and the strongest free radical scavenging and antioxidant activities. Furthermore, MGEB could significantly protect against CCl_4_ induced acute liver injury via control of oxidative stress* in vivo*. In addition, ten proanthocyanidins were isolated from MGEB, and six of them are firstly reported from this plant. Taken together, these findings would be beneficial for developing and utilizing the bark of* M. glyptostroboides*. It also suggested the potential of the proanthocyanidins-enriched fraction as low-cost antioxidant for use in the treatment of various oxidative stress-related diseases.

## Figures and Tables

**Table 1:** The contents of total phenolic, flavonoid, and proanthocyanidin in the extracts and fractions from the bark of *M. glyptostroboides. *

Samples	Yield of extract (%)	Total phenolics (mg GAE/g)	Total flavonoids (mg RE/g)	Total proanthocyanidins (mg PE/g)
MGW	6.9	184.8 ± 0.4^e^	317.3 ± 2.8^e^	26.8 ± 1.6^e^
MGE60	10.1	305.2 ± 1.3^c^	433.5 ± 2.8^b^	348.9 ± 2.6^c^
MGE95	7.0	384.6 ± 0.5^b^	356.7 ± 2.8^c^	404.6 ± 12.6^b^
MGEA	1.4	50.2 ± 3.1^f^	40.6 ± 4.2^f^	1.5 ± 0.1^f^
MGEB	3.4	716.7 ± 8.2^a^	823.4 ± 5.6^a^	627.5 ± 8.7^a^
MGEC	0.4	274.4 ± 0.6^d^	339.0 ± 2.8^d^	56.3 ± 1.1^d^

Values of contents are mean ± standard deviation of three replicate analyses. Means with different letters (a–f) within the same column differed significantly (*P* < 0.05) and are arranged in the following contents descending order: a > b > c > d > e > f.

**Table 2:** Relationship between free radical scavenging, antioxidant activities, and the content of total phenolic, flavonoid, and proanthocyanidin of the extracts and fractions from the bark of *M. glyptostroboides. *

	*R*		
	Total phenolics	Total flavonoids	Total proanthocyanidins
DPPH radical scavenging capacity	0.9127	0.9114	0.9311
Hydroxyl radical scavenging capacity	0.8244	0.7679	0.9242
Superoxide anion radical scavenging capacity	0.8710	0.8251	0.9626
Total antioxidant capacity	0.9319	0.9215	0.9430
Lipid peroxidation inhibitory activity	0.4521	0.4665	0.6941
Fe^2+^ chelating activity	0.6932	0.7387	0.7662

The contents of total phenolics, flavonoids, and proanthocyanidins were expressed as gallic acid equivalents (mg GAE/g sample), rutin equivalents (mg RE/g sample), and proanthocyanidin equivalents (mg PE/g sample). The DPPH, hydroxyl, superoxide anion radical scavenging capacity and total antioxidant capacity were calculated as gram ascorbic acid equivalents per gram sample (g ascorbic acid eq./g). The lipid peroxidation inhibitory activity and Fe^2+^ chelating activity were calculated as half inhibitory concentration (IC_50_) value. Each regression line and correlation coefficient (*R*) were analyzed by the correlation and regression program in Microsoft Office Excel.
